# Biomimetic Strategies for Sensing Biological Species

**DOI:** 10.3390/bios3010089

**Published:** 2013-02-06

**Authors:** Munawar Hussain, Judith Wackerlig, Peter A. Lieberzeit

**Affiliations:** Department of Analytical Chemistry, University of Vienna, Waehringer Strasse 38, A-1090, Vienna, Austria; E-Mails: munawar_arif@hotmail.com (M.H.); judith.maehner@univie.ac.at (J.W.)

**Keywords:** biomimetic strategies, molecular imprinting, polymer affinity materials, membrane mimics, biosensing

## Abstract

The starting point of modern biosensing was the application of actual biological species for recognition. Increasing understanding of the principles underlying such recognition (and biofunctionality in general), however, has triggered a dynamic field in chemistry and materials sciences that aims at joining the best of two worlds by combining concepts derived from nature with the processability of manmade materials, e.g., sensitivity and ruggedness. This review covers different biomimetic strategies leading to highly selective (bio)chemical sensors: the first section covers molecularly imprinted polymers (MIP) that attempt to generate a fully artificial, macromolecular mold of a species in order to detect it selectively. A different strategy comprises of devising polymer coatings to change the biocompatibility of surfaces that can also be used to immobilized natural receptors/ligands and thus stabilize them. Rationally speaking, this leads to self-assembled monolayers closely resembling cell membranes, sometimes also including bioreceptors. Finally, this review will highlight some approaches to generate artificial analogs of natural recognition materials and biomimetic approaches in nanotechnology. It mainly focuses on the literature published since 2005.

## 1. Introduction

One of the driving forces of chemistry is to generate novel materials with improved properties leading to applications that cannot be realized by natural ones. One could regard the advent of plastics as the starting point of this development. Whereas in the beginning, the main fascination arose from being able to generate unprecedented materials, the aspect of studying natural structures as a model for technologically processed materials has been gaining increasing importance. This is perfectly reflected in the following statement: “The inspiration from nature is expected to continue leading to technological improvements and the impact is expected to be felt in every aspect of our lives” [1]. One of the reasons for this is that nature has developed a huge number of very diverse materials by employing comparably few simple building blocks. In most cases, biological “materials” or “structures” are based on fibers that allow for the construction of functional hierarchies [2]. Another important class of materials comprises all types of receptors and recognition elements. In both cases, materials science and chemistry increasingly focus on generating artificial matrices inspired by nature, thus using the biomimetic approach. Within this review, we will focus artificial receptor strategies and their possible applications in chemical and biological sensing. Designing sensors for large (bio)species, such as bacteria, viruses, proteins and cells, is currently developing towards a mature discipline that includes novel approaches depending on the nature and the size of the prospective analyte species. Generally speaking, so far the strategies leading to biomimetic detection of biospecies can be separated into the following categories:

(1) Molecularly imprinted polymers (MIP)
(2) Membrane mimics and self-assembled monolayers (SAM)
(3) Applying peptides, DNA enzymes and natural receptors in artificial environments
(4) Composites, nanoparticles (NPs), nanostructured materials and quantum dots (QDs)

Molecular imprinting actually aims at designing “artificial receptors” or “artificial antibodies” that show biological functionality, despite consisting of a fully artificial, synthetic matrix. Binding to the target analytes occurs by the same non-covalent interactions as in biological systems, but still the backbone is completely different. In the case of membrane mimics, two different strategies are currently followed: the first uses artificial polymers that either repel biospecies or attract them and hence, establish affinity layers for biosensors. These are sometimes combined with natural or nature-analogous receptor/indicator materials to make use of specific interactions in an artificial environment and thus, stabilize the receptor of interest. The second one aims at mimicking natural cell membranes, e.g., with Langmuir–Blodgett techniques and immobilizing (natural) receptors within them. Compared to polymers, this should immobilize the respective target receptor in an environment that is much closer to its original one.

Artificial peptides, DNAzymes, *etc*. aim at focusing on the functional part of a biomolecule and reproducing only this functional part to make synthesis simpler. Nanoparticles and nanostructures themselves are not necessarily biomimetic, but, in combination with different receptor strategies, they open the way for novel biosensing approaches. Within this review, we will highlight sensor strategies falling into these categories and discuss the different aspects of recognition within the systems.

## 2. Molecularly Imprinted Polymers (MIP)

Molecular imprinting is a very straightforward technique to achieve artificial receptors based on highly cross-linked polymers for a wide range of analytes in almost any dimensions [3,4]. In designing those, one has to take into consideration the template, functional monomers, cross-linking monomers, polymerization initiator and the polymerization format [5]. MIPs are widely applicable and have been reported in such different fields as analysis, sensors, extraction and pre-concentration of components. For instance, protein MIPs are a promising tool for producing “artificial antibodies” for recognition. They have been achieved with different material morphologies, such as nanofibers, nanoparticles, hydrogels, thin films of polymers and monoliths by using whole-protein and epitope-imprinting techniques [6]. Their high potential for chemical sensing is given by their inherent re-usability, long-term stability and shelf life, resistance to harsh environment, ruggedness, and low cost.

After a groundbreaking paper by Alexander and Vulfson [7], the literature to date contains epitope or surface-imprinting strategies for different kinds of protein sensors. Additional promising approaches include implementing surface MIP for cells (e.g., yeast), bacteria, virus or blood. To complement the approach, artificial “plastic” copies have also been developed to create standardized template substrates.

**Figure 1:**
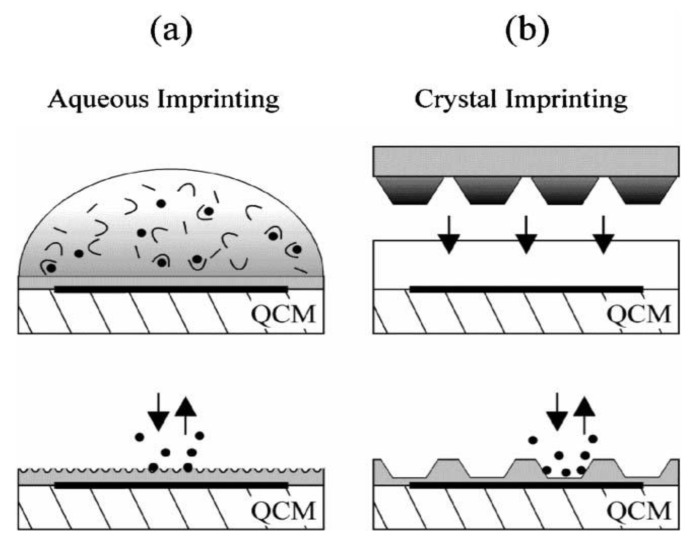
Surface-imprinting strategies on pre-coated QCMs. (**a**) Aqueous monomer solution containing the template is dripped onto the transducer surface for self-organizing receptor sites on the thin film surface. (**b**) A stamp with densely packed trypsin crystals is pressed into the pre-polymerized coating, templates are removed after polymerization. Reprinted with permission from [10], © 2006 The Royal Society of Chemistry.

Firstly, let us regard “straightforward” protein imprinting: human immunodeficiency virus type 1 (HIV-1) related protein (glycoprotein 41, gp41), for instance, has attracted scientific interest, because it is the transmembrane protein of HIV-1 and plays a crucial role in membrane fusion between individual virions and T cells during infection. As such, it plays an important role in the efficacy of therapeutic intervention, because it indicates the extent of HIV-1 disease progression. By implementing the epitope imprinting strategy—where only a substructure of the analyte of interest is used as a template—Lu *et al.* [8] developed a biomimetic sensor to detect gp41. They used quartz crystal microbalance as a transducer and employed dopamine as the functional monomer. They polymerized it in the presence of a synthetic peptide consisting of 35 amino acids corresponding to position 579–613 of the gp41 sequence. It turned out, that the hydrophilic MIP shows substantial affinity towards the target analyte. The dissociation constant (K_d_) of MIP for the template peptide was similar to that of monoclonal antibodies, namely 3.17 nM, calculated through Scatchard analysis. A limit of detection (LoD) of 2 ng/mL was achieved and practical performance was tested on real samples of human urine with satisfactory results. According to the authors, these LoD of HIV-1 gp41 were comparable to the reported ELISA method. On the basis of high its hydrophilicity and biocompatibility, dopamine excels over other functional monomers for this application. Furthermore, this simple epitope method can be adapted to other biomolecules. A further diagnostically important protein is myoglobin, which, among others, can be utilized as a cardiac marker. Rather than going for conventional epitope imprinting, Liao *et al.* [9] presented a surface imprinting strategy. For this purpose, they synthesized the Myo-MIP and established a mass spectrometry-based profiling system for assessing its selectivity compared to other proteins, such as histidine-rich glycoprotein, immunoglobulins, proapolipoprotein, and leech-derived tryptase inhibitor. Whereas the Myo-MIP indeed proved selective, the corresponding NIP (non-imprinted polymer) did not show any sensor signal. Generally speaking, surface imprinting is a versatile technique for protein imprinting: Hayden *et al*. [10], for instance, used 10 MHz QCM as transducers for MIP employing amorphous, crystalline and solubilized trypsin, respectively. The different strategies are summarized in Figure 1.

Sensors reached detection limits of 100 ng·mL^−1^ enzyme and response times of a few minutes. The highest sensitivity was achieved in the case of solution-based imprinting with native trypsin: this system could differentiate denatured trypsin from the native form of enzyme. As already mentioned above, in the case of entire cells, so-called “plastic” copies of the original cells are promising templates for imprinting. For instance, *Saccharomyces cerevisiae*—baker’s yeast—undergoes a range of growth stages, each of which may be recognized biomimetically. Seidler *et al*. [11] produced such artificial plastic copies of natural yeast cells via a “double imprinting” approach. In the first step, a MIP is generated from the native biospecies, from which another surface MIP is cast. The resulting “Master stamp,” e.g., mimics native yeast cells in G0 and early S phase. By replacing the natural template with its artificial analog, sensor synthesis can be standardized. The sensor characteristics of the resulting MIP were similar to those imprinted with native yeast cells. Further improvement is possible by applying electronic nose approaches in parallel: a multichannel quartz crystal microbalance (MQCM) containing four electrodes was optimized for the mass sensitive measurement in liquids. It inherently enables the simultaneous detection of four analytes, ultimately yielding a monitoring system for biotechnology and process control. In a similar approach, Jenik *et al*. [12] fabricated “plastic” yeast cells and used them as a stamp for surface imprinting. The resultant layers can differentiate between *S. cerevisiae *and *S. bayanus, *which strongly corroborates the claim that the functional pattern of the initial yeast cells is correctly reproduced within the polymeric matrix; both native and “plastic” cells yielded the same sensitivity while selectivity exceeded a factor of three in the second case.

MIP can withstand harsh environments and yet retain recognition abilities for the detection of species in complex media. In this connection, Hayden *et al.* [13] further extended surface imprinting to mammalian cells: MIP showing erythrocyte specific interactions have been applied for blood group (BG) typing of the main ABO antigens. By using vinyl pyrrolidone MIP, these ideas could be employed for the development of a recognition system for erythrocytes and ABO blood group typing. Despite higher flexibility of the template cells, MIP layers yielded threefold selectivity and negligible unspecific effects in differentiation of the sub groups A1 and A2. Utilizing “plastic blood cells” resulted in increased robustness of the final MIP as compared to native cells [14].

**Figure 2:**
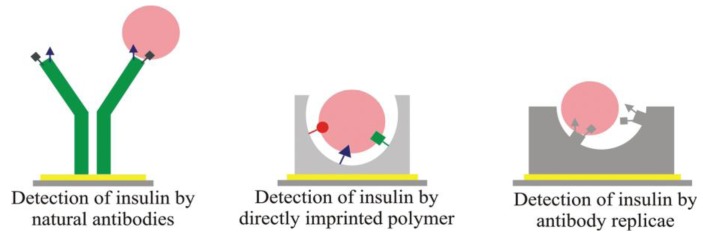
Different way opted for comparative studies in double imprinting. Reprinted with permission from [15], © 2012 American Chemical Society.

Double imprinting approaches can also be applied to literally generate “plastic antibodies” that are inherently robust and selective. For instance, Schirhagl *et al.* [15] developed MIP layers to detect insulin. Additionally, they compared the binding properties of natural anti-insulin antibodies with the insulin surface MIP and achieved similar selectivity between insulin and glargin, but strongly increased sensitivity on the double MIP. Figure 2 depicts the different way opted for during these comparative studies.

Of course, such double-imprinting techniques are especially interesting for generating antibody replica. If immunoglobulin is applied as a template in the MIP, and the resulting substrates as stamps in a second imprinting procedure, this leads to “positive images” of natural antibodies in the polymer. Inherently, such double-imprinted layers can be produced in bulk for industrial processing and are more robust as compared to natural counterparts. Additionally, cost-efficiency increases, because the templates are required only in the first step. MIPs have also been proposed for cell surface proteins, such as wheat germ agglutinin (WGA) lectin, a model compound for interactions between viruses and cells. Wangchareansak *et al. *[16] developed WGA surface MIPs that are stable at room temperature and neutral pH for real-time measurements in aqueous media yielding reproducible results for at least three weeks. In this case, the MIP obviously functions as “crystallizing nuclei” for the protein while NIP disfavors WGA adsorption yielding positive frequency indicating anti-Sauerbrey effects on the QCM.

When comparing MIP-based and natural recognition materials with one another, the following conclusions can be drawn: typically. the selectivity of MIP is in the same order of magnitude as that of natural antibodies or ligand–receptor interactions, or they may even reach it. Mass sensitive measurements revealed that in the case of proteins, MIP functions as a “condensation nuclei”, thus, those systems feature a strongly increasing sensitivity. BET analysis of the WGA MIP mentioned above revealed that the sensor signal at 160 µg/mL WGA corresponds to an average of ten molecular layers on the MIP, whereas affinity to NIP is low. However, the overall sensitivity of any given biosensor is determined by the combination of the recognition material and the respective transducer.

**Figure 3:**
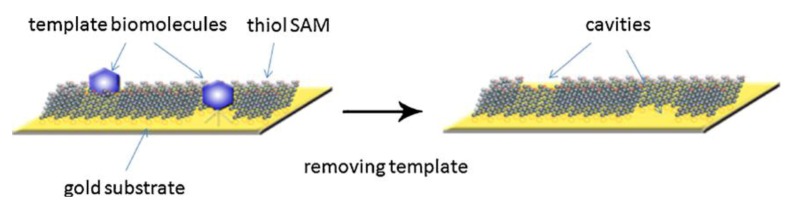
Principle of the method. Formation of biomolecule-templated thiol self-assembled monolayers (SAM), and the subsequent removal of the template molecules to create recognition sites in the SAM matrix. Reprinted with permission from [19], © 2010 Elsevier.

A different approach suggests synthesizing MIP membranes have been reported based on self-assembly to more closely mimic natural functionality, a huge amount of which actually happens in (cell) membranes. For example, Zhao *et al.* [17] used such an approach to generate polyacrylamide-based MIPs for lysozyme that reach adsorption equilibrium 16 times faster compared to previously prepared lysozyme MIP [18]. However, their strategy, in a way, can be regarded as rather “classical”, as they coated a thin film onto the substrate and covered it with Mylar, followed by polymerization at room temperature for 24 h under mild reaction conditions, initiated without heating and ultraviolet radiation to prevent denaturing of lysozyme. In this manner, they could make sure that the template does not change its conformation during polymerization. Wang *et al.* [19] further developed the process and achieved detection of cancer biomarkers and other proteins by synthesizing surface-imprinted, self-assembled monolayers. For that purpose, the authors co-adsorbed hydroxyl-terminated alkane thiols and template biomolecules on gold-coated silicon chips, making use of the covalent bond between the thiol molecules and the gold surface leading to self-assembled monolayers. The biomolecules are removed from the surface which ultimately creates “footprint” cavities in the monolayer matrix. In Figure 3, the principle of the described method is demonstrated. Re-inclusion of biomolecules into the SAM on the sensor chip can be detected potentiometrically. Generally speaking, this leads to very rapid sensor responses and comparably high sensitivities, because electroanalytical techniques that require direct contact between the analyte and the template become realistic. The group of Gauglitz [20] have developed a biomimetic sensor that allows quantification of autoantibodies related to the antiphospholipid syndrome (APS). The substantial challenge in this case is to overcome problems in establishing a reliable recognition element for assessing the target marker against β_2_-glycoprotein-I (β_2_GP-I): β_2_GP-I binds to negatively charged membranes and exposes the correct epitopes only in this bound state. Otherwise, the binding sites are hidden within the protein, which makes immunoassays with the whole protein useless. To provide an environment comparable to the physiological conditions in the human body, the authors co-immobilized a multilayer assembly of covalently attached polymeric DCPEG/PEG (lipid anchor) and a liposome membrane on the transducer. Quantification of anti-β2GP-I antibodies and calibration of the sensor chip in buffer was done using reflectometric interference spectroscopy. This strategy can be extended to distinguish between healthy and ill individuals within routine clinical diagnostics. Furthermore, it is an impressive example for the fact that in bioanalysis not only the target analyte plays a role, but also its environment. In this way, the sensor surface mimics not “only” an antibody or binding site, but also the membrane structure necessary to ensure biological activity.

## 3. Polymer Thin Films, Self-Assembled Monolayers (SAM) and Membrane Mimics

Membranes play a key role for life, as they are interfaces between cells and their surroundings. As such, they are also interesting starting points for biomimetic sensor development. Here, two seemingly contradictory goals are of main interest: on the one hand—and as mentioned already in the previous section—the design of artificial receptors, and on the other hand, effectively preventing nonspecific protein adsorption from real-life media (e.g., blood serum), which is absolutely key for implanted medical devices. Addressing this issue, Vaisocherova *et al.* [21] applied functional zwitterionic poly(carboxybetaine acrylamide) (poly CBAA) that inherently repel protein adsorption and immobilized specific antibodies to this surface. Such an assembly can be regarded as a mimic of a receptor in an otherwise non-functional membrane. Combining the high affinity and selectivity of the antibody and the antifouling layer hence, leads to a highly (mono)functional receptor with minimized non-specific effects. On surface plasmon resonators (SPR), the setup leads to highly selective and sensitive detection for *in vivo* diagnostics without showing any significant response to the anti-Salm-functionalized surface (a rabbit polyclonal antibody to *Salmonella* species). Astonishingly, high selectivity and sensitivity (<3 ng/cm^2^ for undiluted blood plasma) was achieved for an activated leukocyte cell adhesion molecule (ALCAM, CD 166), a potential carcinoma biomarker. Such combinations of artificial and natural materials have also been reported by other groups. Henry *et al*. [22], for instance, reported low density electrochemical DNA sensor arrays for pharmacogenomics and theranostics, thereby emphasizing analysis speed and low costs. Compared to “natural” genosensor arrays detecting individual nucleotides polyethylene glycol (PEG), co-immobilization strategies for DNA substantially increase ruggedness and shelf life of the sensors. This was shown by breast cancer marker estrogen receptor as the model system for detection. The resulting systems are both electrochemically sensitive and resistive against fouling as they combine electron permeability and a hydrophilic surface. Electrochemical testing, including electrochemical impedance spectroscopy validated by surface plasmon resonance, revealed that a combination of bipodal aromatic PEG alkanethiol and the respective receptor in a ratio of 100:1 reaches detection limits of 0.17nM without interferences from non-specific binding.

When using pure polymer films, *i.e*., not in combination with natural materials, the aim can also be facilitating the formation of adhered biolayers rather than preventing them. One example for this strategy has been reported by Mueller *et al.* [23], who modified QCM electrodes with polyethylene thin films to facilitate the coagulation of blood proteins on the sensor surface and related it to the coagulation time of human whole blood samples. The polymer in this case “simulates” an extracorporeal surface and hence allows for the exact determination of the onset of blood coagulation. Inherently, the approach shall lead to a diagnostic monitoring system that automatically supplies sedated patients with the correct dose of anticoagulants during surgery. Using the same procedure, Sinn *et al*. [24] applied ultrathin films of polyethylene oxide-polypropylene oxide co-polymers NCO-sP(EO-stat-PO) for quantification of fibrinogen adsorption in protein resistance measurements. In such types of real-time studies, the unspecific adsorption of plasma proteins and blood cells hamper the reliability of implantable blood sensors. Using a polymer coating, the unspecific absorption could be reduced by a factor of 80% compared to uncoated electrodes of the QCM.

Polymer films can also function as “affinity receptors” themselves: Ibrišimović *et al*. [25] for instance deposited biodegradable layers made from Poly (lactic-co-glycolic acid) (PLGA) onto sensor surfaces that are broken down by lytic enzymes released from decaying cell material. This results in a thickness change that is visible even with the naked eye. Such an approach is e.g., highly interesting for detecting microbial spoilage in foodstuff and thus to indicate quality in real time by bringing the sensor into direct contact with the (probably microbially contaminated) sample. The seminal advantages of such sensor prototypes are low-cost production and processing potential and thus inherently integration into product packaging. Of course, such systems are neither reversible, nor stable on the long term, but this is not required in such an application scenario. A similar approach has been used by Meir *et al. *[26], who constructed sensing layers based on agarose-embedded, chromatic Langmuir-Schaefer phospholipid/polydiacetylene films. During bacterial growth on their surfaces, the sensors substantially change their colors and yield strong fluorescence due to amphiphilic compounds excreted from the bacteria. With such a setup, for instance, Gram-negative and Gram-positive bacteria can be detected and physiological fluids and food (e.g., meat) can be scanned for bacterial contamination. Applying a somewhat different strategy, the same group [27] used glass-supported films of lipids and polydiacetylene for visual detection and colorimetric fingerprinting of bacteria. This is achieved through the affinity of bacteria and their secreted compounds towards phospholipid bilayers. Within the biomimetic membrane, the polydiacetylene is used as a chromatic reporter sandwiched between two lipid monolayers. Basically, this can be regarded as a cellular membrane containing a reporter/indicator compound within. The advantages include straightforward and cost-effective synthesis of films as well as ruggedness and long shelf lives. The system allows for detection limits down to 10^6^ particles/mL after short incubation and can be monitored by naked eye or readily available scanners. Although, detection limits are a few order of magnitudes higher than in modern PCR strategies, the technique offers unparalleled simplicity in its approach.

Such straightforward receptor materials, of course, allow for evaluating novel transducers, e.g., derived from modern nanotechnology as bio(mimetic) sensors. Chang *et al.* [28], for instance, reported on a silicon nanowire field-effect transistor (SiNW-FET) coated with polyvinyl chloride (PVC) membranes containing valinomycin (VAL), a well-known cryptand for detecting potassium ions. Although, in principle, a well-known approach, miniaturization in this case allows for measuring the ionic efflux from living chromaffin cells. The sensor characteristic for the setup shows a dynamic range from 10^−6^ M to 10^−2^ M. In a related, yet completely different approach, Yang *et al.* [29] synthesized novel bio-mimicking graphene films and deposited them on textured nickel substrates. On those, they reproduced the surface texture of lotus leaves and cell-like structures, e.g., by duplication and electroplating. Possible application scenarios for such systems are as nano-electronics, electrodes, capacitors, batteries and culturing of electrically excitable cells.

Generally speaking, electrochemical impedance spectrometry is gaining increasing importance in the detection of bioanalytes down to viruses [30] due to its inherently non-invasive approach. This has also triggered fundamental interest in conducting polymers. For example, Polypyrrole (PPy) can be electrosynthesized in the presence of anionic species, including net-negatively charged biological molecules such as proteins and polysaccharides. According to Ateh *et al*. [31], chloride-loaded PPy films on gold substrates can be applied for studying a range of skin-derived cells. The reason for this is that the change in cell-induced impedance depends on cell density and type. Compared to bare gold electrodes, detection limits are lower due to the more biocompatible surface and keratinocyte confluence is reached faster. However, also inorganic coatings can serve the same purpose: Zhu *et al. *[32] showed that *Salmonella typhimurium *can be detected *in situ *with highly selectivity at 90% relative humidity by using a lead zirconatetitanate (PZT)/gold-coated glass cantilever. Its sensitivity was found to be 1 × 10^3^ and 500 cells/ml in 2 mL of liquid with a 1 and 1.5 mm dipping depth, respectively. Such a limit of detection is more than one order of magnitude lower than that of the commercial Raptor sensor.

**Figure 4:**
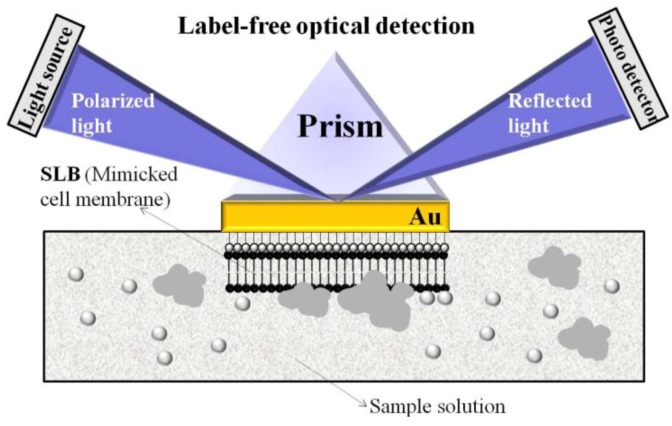
Illustration of biomimetic SLB/SPRS set-up for the observation of membrane behavior. Reprinted with permission from [34], © 2009 IEEE.

Self-assembly is one of the fundamental driving forces of life that leads, for example, to the phospholipid bilayers constituting cell membranes. The Langmuir–Blodgett technique is a way to build up artificial membranes. It makes it possible, for instance, to immobilize lipid bilayers (LB) that themselves can stabilize biomolecules to achieve biomimetic “cell membranes.” In contrast to the methods previously mentioned, the biomolecule is therefore immobilized in an artificial membrane that more closely resembles a natural one, as compared to a polymer thin film. Using this approach, Jiao *et al.* [33] demonstrated the use of non-inhibitory antibodies employed in a luminal derivative LB film for the reagentless detection of electrochemiluminescence (ECL) and antibody insertion. For model studies, choline oxidase activity has been detected, because catalytically H_2_O_2_ produced *in situ* can trigger ECL reaction in the sensing layer. In another approach of using supported lipid bilayers (SLB) mimicking natural structures, Choi *et al.* [34] demonstrated a novel biosensor for monitoring the behavior of cell membrane linking proteins *in vitro* by label-free surface plasmon resonance spectroscopy (SPRS). Biomimetic sensor chips were fabricated by the fusion of unilamellar lipid vesicles on a hydrophilic Au surface for SPRS. This setup enables real-time measurements of protein aggregation. Figure 4 illustrates the proposed biomimetic SLB/SPES design for such measurements.

The respective membrane behavior was analyzed by investigating specific types of Cu/Zn-superoxide dismutase (SOD1) species, which are linked to neurodegenerative disease. In an unusual manner, SOD1 aggregates degenerate membranes closely related with aberrant characteristics of conformationally disordered and/or mostly aggregated proteins. The presented biomimetic sensor build-up can be further applied for the development of biomarkers to analyze different injured cells.

## 4. Artificial Analogs of Natural Species

In recent years, peptide-based sensors have been developed, including a range of different strategies. One of these can be regarded as biomimetic, namely synthesizing peptides that are designed to make use of well-known interactions between a single or a few amino acids and the respective target compounds. However, selecting them can be difficult, when the target molecule is small and/or it considerably changes its conformation upon immobilization [35]. Other biomimetic (artificial) strategies have been reported based on DNA and natural receptors. All of those will be discussed in this section.

The detection of circulating tumor cells (CTCs) is clinically important for diagnosis and prognosis of cancer metastasis. Separation and detection of these cells are challenging due to extremely low quantity in patient blood (as low as 1 in 10^9^ hematologic cells). To cope with the challenge, Myung *et al. *[36] employed biomimetic surfaces functionalized with P- and E-selectin and anti-EpCAM that produce different responses in HL-60 (used as a model of leukocytes) and MCF-7 (a model of CTCs) cells. The two cell lines experienced a different extent of interaction with P-/E-selectin and anti-EpCAM at a shear stress of 0.32 dyn/cm^2^. Hence, they can be separated from one another and detected finally by CTC devices that offer enhanced selectivity and sensitivity without involving complex fabrication processes. These differences are governed by the fact that the ratio of cell rolling and stationary binding on the surface depends on the respective immobilized artificial peptide. When designing artificial recognition sequences, applying molecular modeling of the actual interaction center usually substantially improves the overall experimental time, as candidate receptors can be selected “*in silico*.” An example for this is the rapid and direct QCM detection of the mussel heat shock protein HSP70 in crude extracts of the mussel mantle by artificial heptapeptides [37]. After finding optimal amino acid sequences for interacting with the hapten of HSP70, they immobilized them on QCM and compared the sensor results obtained with those of HSP70-specific antibodies. Both methods yield similar selectivity and sensitivity on the sensors, which again very impressively underpins the possibilities of biomimetic approaches.

**Figure 5:**
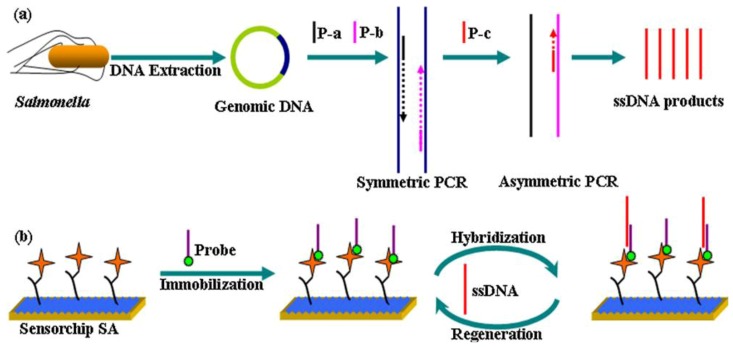
Schematic illustration of the strategy for *Salmonella* detection. (**a**) DNA extraction from *Salmonella* cells and amplification using the second step-asymmetric PCR (**b**) Probe immobilization and interaction with PCR amplicons. Reprinted with permission from [38], © 2012 Elsevier.

An example for a biomimetic DNA-based system has been presented by Pelossof *et al.* [39]. They coated a hemin/G-quadruplex DNAzyme mimicking horseradish peroxidase (HRP) onto a gold electrode as electrocatalytic label. Its bioelectrocatalytic properties make it possible to develop electrochemical sensors which lead to activity of glucose oxidase and biosensors for DNA detection. Despite the fact, that the electrocatalytic activity of HRP-mimicking DNAzyme is lower than that of native horseradish peroxidase, the detection limits for DNA target was found to be 1 × 10^−12^ M, while a limit for AMP of 1 × 10^−6^ M was reached. Extending DNA biomimetic sensors, Zhang *et al*. [38] developed a new method of DNA biosensors for label-free, high-sensitive, specific and rapid sensing of *Salmonella*. They designed a biotinylated single-stranded oligonucleotide probe targeting a specific sequence in the invA gene (*Salmonella*-specific gene) of that species and immobilized it onto a streptavidin-coated dextran sensor surface. Employing surface plasmon resonance (SPR), detection limits of 0.5 nM were obtained with linearity from 5 to 1,000 nM. *Escherichia coli* and *S. aureus *yield no significant signal in selectivity pattern studies. Figure 5 illustrates the described strategy for *Salmonella* detection.

## 5. Composites, Nanoparticles (NPs), Nanostructured Materials and Quantum Dots (QDs)

Molecular self-assembly processes are crucial for generating new functional materials with suitable prosperities for sensor development. This concept is biomimetic to its core, as nature has, for example, constructed thousands of nanostructures from 20 amino acids [40]. Chemical and topographic substrate surface patterning is considered to be a promising tool for regulating cell functions. Lim *et al. *[41] discussed the relative role of scale and pattern of chemically and topographically patterned surfaces for regulation of cell behavior. Establishing spatial cell-adhesive molecular organization, chemical patterning can be achieved. Such patterns allow regulating different cell functions based on their scale. Additionally, in the case of topographic patterns, the micro- or nanoscale governs specific cell reactions. Nanometer-scale structures, such as nanotubes, nanowires, nanorods, nanospheres, nanorings, nanoribbons, nanocomb, nanoflowers, nanofibers, nanoparticles, and nanocomposite materials, can be applied at different stages for the detection of biomolecules. These nanostructured materials are biocompatible, non-toxic, have high specific surface area, chemical and thermal stability, electro-catalytic activity and fast electron communication properties leading to higher sensitivity, selectivity, linearity, fast response and reproducibility of the analytical devices [42,43,44].

**Figure 6:**
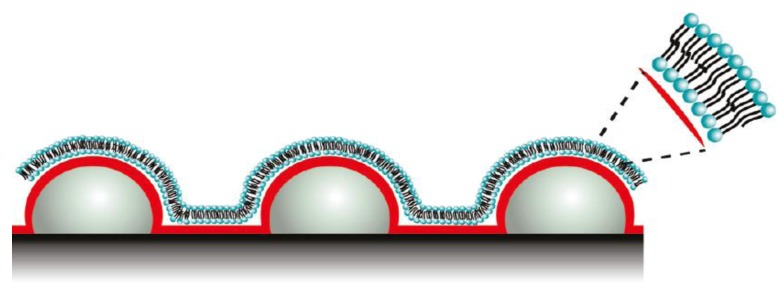
Schematic representation of lipid bilayers used for protein binding. Reprinted with permission from [45], © 2011 American Chemical Society.

Motivated by biological processes, Sundh *et al. *[45] developed novel biomimetic membranes generated at nanostructured sensor substrates with controlled curvatures. Such template-supported lipid bilayers (SLBs) with controlled curvature allow for lipid sorting, phase separation, and protein binding. The SLBs were generated by vesicle adsorption followed by rupturing their structures. SLBs contain increased surface area due to underlying nanostructured surfaces with decreased radii of curvatures (ROC) confirmed by excess mass loading on QCM-D, and excess total fluorescence intensities. Figure 6 depicts the lipid bilayers used for phase separation and protein binding. Lipid layers with differing lipid compositions and ROCs are inherently applicable for studying cell membrane related processes in sensor applications using QCM-D.

**Figure 7:**
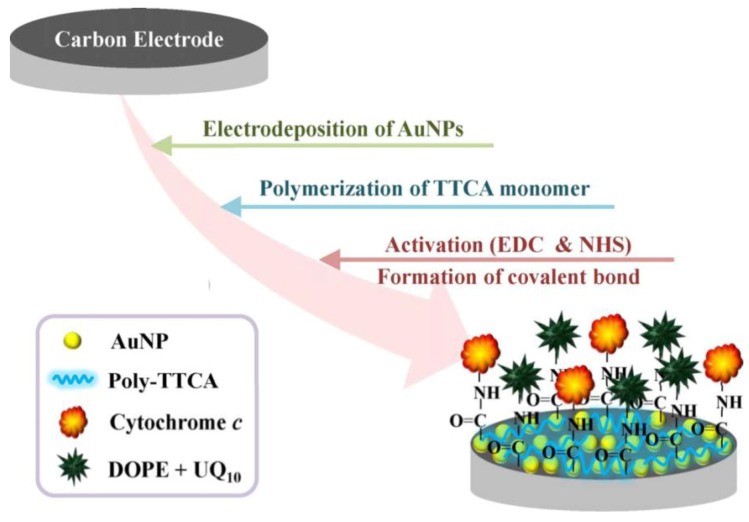
Schematic diagram for fabrication of a biomimetic membrane. Reprinted with permission from [46], © 2009 IEEE.

The outstanding effect of nanoparticles (NP) has been demonstrated by Lee *et al.* [46] through developing an electrochemical sensor for NADH. As sketched in Figure 7, they modified a carbon electrode surface with gold nanoparticles (AuNPs) and electrodeposited a conjugated polymer (5,2′:5′,2′′-terthiophene-3′-carboxylic acid, TTCA), which acts as substrate for an artificial biomembrane. The specific bioaffinity properties were ensured by immobilizing ubiquinone (UQ_10_) onto the modified electrode simultaneously. Using cyclic voltammetry, a sensitivity of −0.04988 μA/mM for NADH with the modified electrode could be achieved.

Similarly, employing chitosan-modified magnetic Fe_3_O_4_ nanoparticles (CMNP) as signal amplifiers, Lin *et al.* [47] reported a cost effective and disposable remote wireless sensor detecting pathogens by utilizing *Escherichia coli* as a target. For this purpose, CMNP were functionalized with a chitosan layer for binding negatively charged *E. coli* via electrostatic attraction. The CMNPs binding to the sensor resulted in increased mass loading ultimately decreasing its resonance frequency without interference by serum proteins. The method can be extended further to other bacteria. For example, slightly varying the NP approach, the immobilized Au nanoparticles were functionalized with single-strand DNA (ssDNA) probes specific to the eaeA gene of *E. coli* O157:H7 [48]. First reports have been made to also combine bio-selective MIP for detecting bovine hemoglobin with magnetic nanoparticles [49]. In a different approach, Saifullina *et al. *[50] demonstrated highly sensitive sensors by using a thick layer of carbon nanotubes for electrochemically monitoring the metabolic activity of encapsulated cells. This setup also involves modification of the electrode surface with a polysaccharide matrix. The strategy can be further employed to a microarray format for efficient analysis of cytocompatibility of soft polymeric materials and antifouling studies.

**Figure 8:**
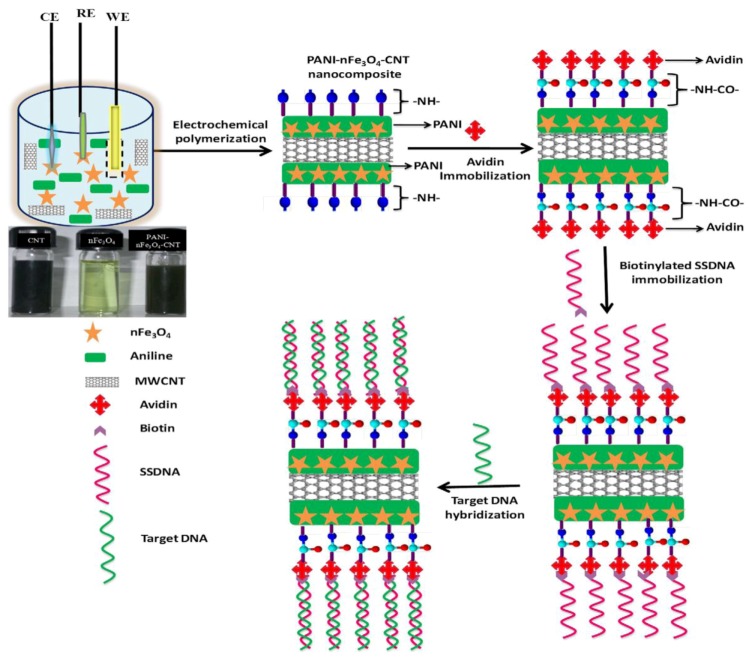
Preparation of PANI-nFe_3_O_4_-CNT nanocomposite and immobilization of biotinylated DNA using biotin-avidin coupling followed by hybridization for bacterial detection. Reprinted with permission from [53], © 2012 Elsevier.

Mesoporous materials constitute another interesting class of substrates for biomimetic sensor setups. For instance, Li *et al.* [51] synthesized an organic mesoporous material consisting of polyacrylamide-P123 (PAM-P123) composite films for binding hemoglobin onto the surface of glassy carbon electrodes. As compared to inorganic mesoporous materials—which are comparably widespread—the PAM-P123 composite film has a better film-forming characteristic. They used this sensor for studying the direct electron transfer of hemoglobin—which can be extended to other redox-active enzymes—and fabricating a sensitive voltammetric biosensor for H_2_O_2_. Combining the above two strategies (*i.e*., nanotubes and composites), Kim *et al.* [52] employed carbon nanotube (CNT)-based epoxy and rubber composites by fabricating a CNT flexible strain sensor for tactile sensing. Such a sensor uses a long fibrous sensor and can measure large deformation and contact information on a structure. The sensor can be regarded as a biomimetic artificial neuron because it bears some similarities with the dendrites of a neuron in the human body. Designing Artificial Neuron Matrix Systems (ANMS) by manufacturing arrays of these neurons allows signal processing for biomimetic engineering applications, e.g., as artificial e-skin with tactile sensing properties. A slightly different approach is described by Singh *et al.* [53]: they designed a nanocomposite of polyaniline (PANI)–iron oxide nanoparticles (nFe_3_O_4_) and multi-walled carbon nanotubes (CNT) coated onto indium tin oxide (ITO) coated glass plate by electrochemical synthesis of polyaniline (see Figure 8). The resulting nanocomposite can be adapted to ultra-sensitive bacterial (*N. gonorrhoeae*) genosensors leading to detection limits of 1 × 10^−19^ M bacteria within 45 s of hybridization time at 298 K.

**Figure 9:**
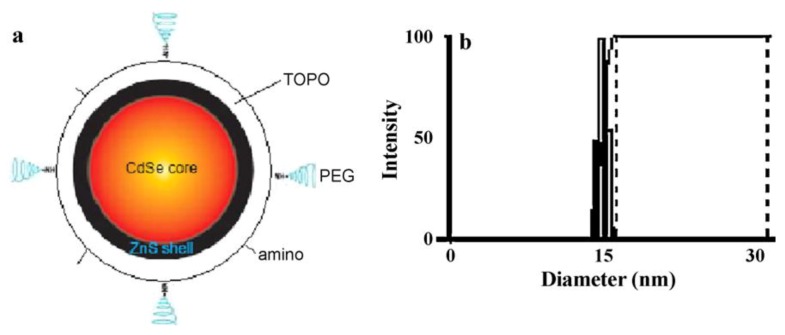
The structure of the amino modified CdSe/ZnS core-shell QD (**a**) and the size distribution of the QDs (**b**) in the aqueous environment of living cells. The averaged diameter of the QD is 15.0 ± 0.8 nm. Reprinted with permission from [54], © 2011 Elsevier.

**Figure 10:**
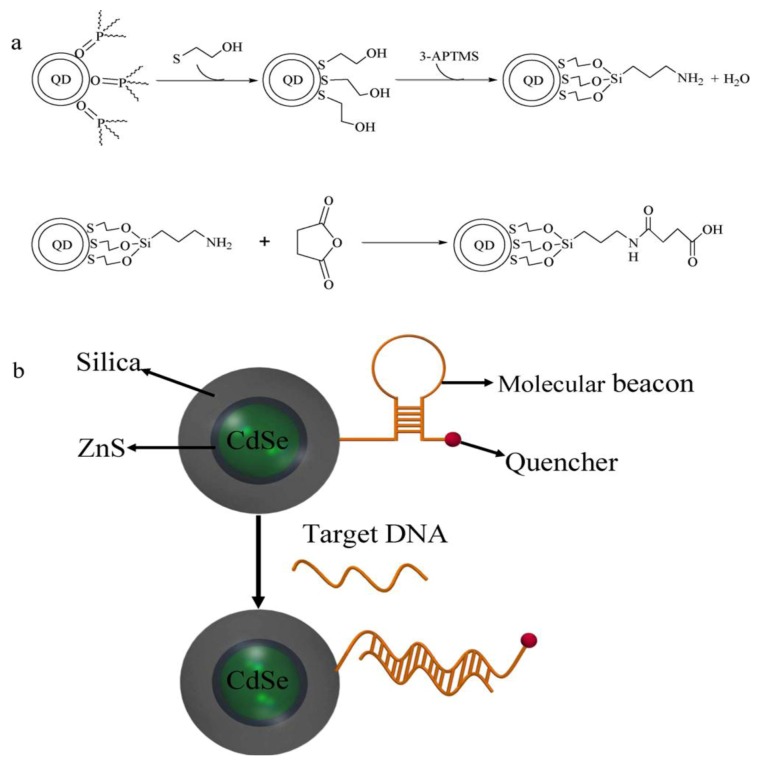
(**a**) Reaction scheme of the silanization and functionalization of QDs (**b**) Schematic of a QD based molecular beacon. Reprinted with permission from [55], © 2011 Elsevier.

Being a comparably new class of nanomaterials, quantum dots (QDs) are considered to be promising matrices for sensor layers. For example, employing QDs as biomimetic material, Huang *et al*. [54] developed a technique for quantitative visualization of the surface charge on biological cells with nano-scale resolution. The QDs were generated by using amino modified CdSe/ZnS nanoparticles (as seen in Figure 9(a)) altered by modular ligands based on poly(ethylene) coupled with amino to enhance their stability and biocompatibility. This modification leads to positively charged QDs with a diameter of about 15 nm (Figure 9(b)). The charge densities of different kinds of cells from normal to mutant have been detected by employing wide-field optical sectioning microscopy for 2D/3D imaging of the QD-labeled cells. The surface charge distribution is important for analysis of structure, function, biological behavior and malignant transformation of cells. Similarly, Wu *et al. *[55] applied QDs for the development of highly sensitive molecular beacon (MB) for DNA sensing. Figure 10 summarizes the functionalization of QDs and the principal setup of the QD based molecular beacon. Each beacon consists of a “smart probe” which quenches the fluorescence in the absence of the target DNA. The onset of fluorescence emission thereby, indicates the presence of target DNA (Figure 10(b)). In this case, the QD approach allows for substantially increased quencher densities, as compared to flat surfaces and thus increased signals. A possible combination of NPs and QDs is demonstrated by Xie *et al.* [56]. Fe_2_O_3_ magnetic nanoparticles and fluorescent quantum dots were embedded together into single swelling poly(styrene/acrylamide) copolymer nanospheres. In this way, fluorescent-magnetic bifunctional nanospheres can be generated. Their approach included the fabrication of smart wheat germ agglutinin (WGA)- trifunctional nanobiosensors (TFNS), peanut agglutinin (PNA)-TFNS and Dolichos biflorus agglutinin (DBA)-TFNS composites that combine high fluorescence yield, magnetic properties and selective detection of *N*-acetylglucosamine, D-galactosamine and *N*-acetylgalactosamine residues on the A549 cell surface. Such biomimetic lectin-modified nano biosensors (lectin-TFNS) can thus be employed for analysis of glycoconjugates on A549 cell surface.

## 6. Conclusions

As can be seen, mimicking biological functionality has become a field of substantial scientific interest and has generated a range of different strategies for actually reaching that goal. Whereas, the idea behind molecular imprinting is to generate as high selectivity as possible in a fully artificial material, polymer thin-film coatings and self-assembled monolayers follow different goals and strategies: first, they are optimized to tune the affinity between cells and surfaces, which can lead both to materials preventing cell adhesion, as well as to ones supporting it. Furthermore, such systems are useful to incorporate and stabilize biological receptors, hence, generating a monofunctional surface containing a specified interaction center.

Compared to the substantial scientific effort, however, such systems are commercially not (yet) very successful. It is difficult to attribute these to concrete reasons. However, the following points definitely do play a role:

- Diagnostics and healthcare, as well as security topics (e.g., detecting illegal drugs) are the main driving forces behind biosensing. Those fields do not necessarily require reversible sensors, but disposable systems. Therefore, they do not require the main advantages of biomimetic materials, which are their ruggedness and re-usability.
- Sensitivity of biomimetic materials reaches, or even exceeds, that of e.g., antibody-based test formats. However, polymerase chain reaction (PCR) has made substantial progress during the last two decades allowing for extremely low detection limits for microorganisms due to its amplification properties.
- The market of diagnostic tools is inherently conservative and thus reluctant to replace well-established techniques with novel ones due to lack of experience or expertise.
- Especially in MIP, studies on batch-to-batch reproducibility and upscaling to (pilot) plant level still have to be done.

In conclusion, biomimetic sensing is still a matter of research at the academic level, rather than commercial development. Replacing—or at least complementing—bioreceptors will thus not be possible in the immediate future. However, the main advantages of artificial systems will make them interesting candidates for measuring applications requiring long-term stability, such as process control or monitoring air/water quality over extended periods of time. Once established in those markets, application in the diagnostic area seems more realistic, as artificial materials offer inherent cost advantages.
